# Clinico-epidemiological study of *Schistosomiasis mansoni* in Waja-Timuga, District of Alamata, northern Ethiopia

**DOI:** 10.1186/1756-3305-7-158

**Published:** 2014-04-01

**Authors:** Nigus Abebe, Berhanu Erko, Girmay Medhin, Nega Berhe

**Affiliations:** 1 Mekelle University, College of Veterinary Medicine, Mekelle, Tigray, Ethiopia; 2 Addis Ababa University, Aklilu Lemma Institute of Pathobiology, Addis Ababa, Ethiopia

**Keywords:** *Schistosoma mansoni*, Intensity, Periportal fibrosis, Waja-Timuga, Ethiopia

## Abstract

**Background:**

Intestinal schistosomiasis, caused by digenetic trematodes of the genus *Schistosoma*, is the most prevalent water related disease that causes considerable morbidity and mortality. Although prevalence of *Schistosoma mansoni* infection has been reported for the present study area, earlier studies have not estimated intensity of infections in relation to periportal fibrosis, which would have been crucial for epidemiological and clinical evaluations. Hence, a community based cross sectional study was conducted from December 2011 to March 2012 to assess prevalence of infection and schistosomal periportal fibrosis in Waja-Timuga, northern Ethiopia.

**Methods:**

In a cross sectional study involving 371 randomly selected individuals, fresh stool samples were collected and processed by the Kato-Katz method and examined microscopically. Ultrasonography was used to determine status of schistosomal periportal fibrosis and to detect hepatomegaly and/or splenomegaly. Serum was collected for assay of hepatic activity. Statistical analysis was performed using STATA 11 statistical soft ware. P-value <0.05 was reported as statistically significant.

**Results:**

The prevalence of *S.mansoni* infection was 73.9%, while the prevalence of schistosomal periportal fibrosis was 12.3% and mean intensity of infection was 234 eggs per gram of stool. Peak prevalence and intensity of *S.mansoni* infection was documented in the age range of 10–20 years. Among the study individuals, hepatomegaly was recorded in 3.7% and splenomegaly was recorded in 7.4% of the study individuals. Similarly, among the study individuals who had definite periportal fibrosis, 5.9% had elevated liver enzyme levels.

**Conclusion:**

The high prevalence of *Schistosoma mansoni* infection and schistosomal periportal fibrosis observed in the study area calls for a periodic deworming program to reduce disease, morbidity and transmission. Preventive chemotherapy complemented with other control measures is highly required for sustainable control of schistosomiasis in the study area.

## Background

Intestinal Schistosomiasis is caused by *S. mansoni* and transmission occurs when the human definitive host skin comes in contact with cercariae of *Biomphalaria* snails. The disease remains one of the highly neglected tropical diseases, which causes high morbidity and considerable mortality in sub-Saharan Africa [1,2].

In endemic areas, children carry the largest burden of the disease characteristics of acute schistosomiasis are rarely seen; however, the adult population carries the chronic form of the disease characterized by periportal fibrosis, hepatomegaly, splenomegaly, portal hypertension, hematemesis and oesopheageal varices [3,4].

Morbidity and mortality due to schistosomiasis are largely due to the consequences of a host T cell-mediated immune response against parasite eggs trapped in the tissue. Antigens released from the egg stimulate a granulomatous reaction involving T cells, macrophages, and eosinophils that results in clinical disease. However, the magnitude of the resulting granulomatous and fibrosing inflammation varies greatly from individual to individual [5], which seems to be influenced to a large extent by the nature of the induced immune response and its effects on granuloma formation and associated pathologies in target organs. In addition, the extent of morbidity differs markedly among individuals of similar ages with similar intensities of infection, both between and within communities: other factors may, therefore, also be involved, including host and parasite genetic differences, nutritional status, exposure to other infectious agents, and maternal infection status [6,7]. The degree of morbidity associated with *S.mansoni* is related to the intensity of infection and other factors. Symptoms and signs depend on the number and location of eggs trapped in the tissues. Initially, the inflammatory reaction is readily reversible. In the latter stages of the disease, the pathology is associated with collagen deposition and fibrosis, resulting in organ damage that may be only partially reversible. In light infection, individuals remain asymptomatic except in infants where bloody stools are seen, in mild infections, bloody stool, abdominal pain and nausea can occur, while in the heavy infections, individuals show lesions of the liver and spleen [8-10].

Ultrasonography is currently the diagnostic tool of choice for detecting pathologic conditions associated with schistosomiasis mansoni such as liver fibrosis and enlargement and dilatation of the portal vein [11,12]. Moreover, it provides sensitive and precise measurements of *S.mansoni*-associated pathologic changes [13].

In Ethiopia, the distribution of schistosomiasis is highly focal and varies from region to region because of several environmental, social and geographical factors [14]. The spread of schistosomiasis in Ethiopia is mainly attributed to water resource development and increasing population movement [15]. Although prevalence of schistosomiasis mansoni has been reported for the current study area [16], community level information on the epidemiology of the infection and schistosomal periportal fibrosis (PPF) is scarce for most endemic foci including the present study area. Therefore, the objective of the current study was to determine the prevalence of schistosomiasis mansoni and schistosomal PPF in Waja-Timuga, District of Alamata, northern Ethiopia.

## Methods

### Study area and population

**Figure 1:**
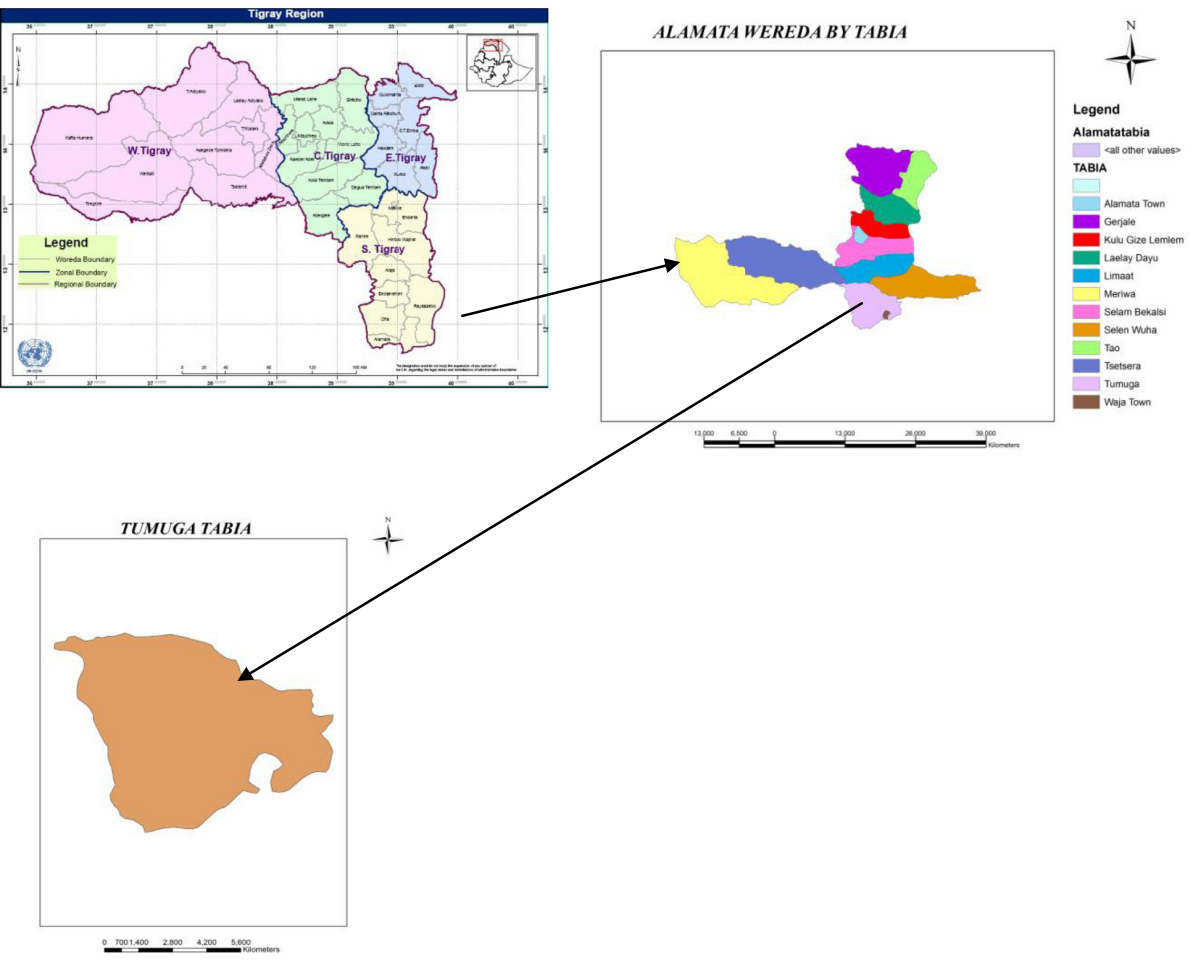
**0 Map of study site**.

The current study was conducted in southern Tigray, Alamata District, Waja-Timuga area, which is located 600 km north of Addis Ababa, the capital city of Ethiopia, and about 185 km south of Mekelle, the capital of the Tigray Regional State (Figure 1). Waja-Timuga has a total population of 11,030 (1051 households) with area coverage of 5985.5 km^2^ and about 10 *tabias* (local administrative units). The area is located between latitude of 12°19’N and longitude of 39036’E at 1470 m above sea level [17]. The inhabitants of the study area earn their living as government employees, farmers and self-employed (merchant, daily labourer etc.).

### Study design, sample size determination and sampling method

This was a community based cross sectional study conducted between December 2011 - March 2012.

Sample size was determined based on the previous prevalence of 67.95% obtained from a study conducted among schoolchildren in Timuga and Waja, northern Ethiopia [18]. A 95% level of confidence (z = 1.96) and 5% margin of error (d) was used. With these assumptions the minimum sample size required for the study was 334 and with 11% allowed for non-respondents, the required sample size increased to 371.

A list of 1051 households obtained from the local administrative units served as the sampling frame. A simple random sampling method was used to select study participating households. Using this method, one-hundred-fifty households from three accessible communities namely; 28 households from Waja, 71 households from Timuga and 51 households from Aroresha were selected. All family members were invited for *S.mansoni* infection examination.

### Stool collection and examination

Fresh stool specimens were collected using pieces of plastic sheets distributed to each of the selected individuals. Specimens were processed for microscopic examination after 24 hrs of smear preparation for evaluation of *S. mansoni* eggs by the quantitative Kato-Katz method using a template delivering 41.7 mg of stool [19].

Two thick Kato-Katz smears were prepared from a single stool sample. The slides were labeled and transported using slide boxes to the parasitology laboratory at The College of Veterinary Medicine, Mekelle University for microscopic examination. Egg counts per slide were multiplied by 24 to convert into number of eggs per gram (EPG) of stool [19].

For quality control, 10% of the slides were randomly selected and re-examined by an experienced laboratory technician who was blinded to schistosome infection status of the individual. Any discrepancies were counter checked before results were recorded.

The intensity of infection was classified according to WHO [20] as light (1–99 EPG), moderate (100–400 EPG) and heavy infections (more than 400 EPG).

### Ultrasonography

Ultrasonographic assessments were performed to detect pathological changes associated with *S.mansoni* infection in individuals who participated in parasitological screening. Using the WHO-Niamey protocol [21], standard ultrasonographic liver scans were performed using Hitachi EUB 405 portable ultrasound equipment (Tokyo, Japan), fitted with a 3.5-MHz convex abdominal probe. All examinations were performed by the same clinician, who was blinded to the schistosome infections status of the individual.

In individuals with image patterns suggestive of PPF, the liver picture was compared with standard images and the corresponding image pattern score was recorded. In addition, assessment of the periportal thickening was made by taking inner to inner and outer to outer portal branch wall thickness (PBWT) measurements of branching portal veins. These measurements were close to branching points of the vessels, and the arithmetic mean differences between outer and inner wall thickness were taken as the individual’s PBWT value. The summation of the image pattern and PBWT scores gave the final PPF grading of each individual. Accordingly, individuals with no/non-specific diffuse echogenic liver pattern associated with minimal wall thickening were classified as having “no PPF”, those with image patterns suggestive of PPF but with wall thickness less or equal to mean ± SD of normal PBWT-for height standard were classified “indeterminate PPF”, and those with definite or advanced PPF by both image pattern and PBWT measurements were classified as “definite PPF” (Additional file 1).

### Serum collection and examination

Five milliliters (ml) of venous blood was collected from individuals whose ultrasound results were suggestive of image pattern PPF. Serum was separated by centrifugation and stored in thermo boxes at -20 ºC in a freezer prior to dispatching to the Aklilu Lemma Institute of Pathobiology, Addis Ababa University. Levels of serum glutamic-oxaloacetic transaminase (SGOT) and serum glutamate-pyruvate transaminase (SGPT) was determined using a photometer (Photometer 5010, Germany) as biomarkers of liver function test. The ranges of SGOT and SGPT numbers may differ slightly depending on the technique and protocols used by different laboratories. Nevertheless, normal reference ranges were routinely provided by each laboratory and printed in the report. Although sources vary on specific reference range values for patients, 10–40 IU/L is the standard reference range for experimental studies [22].

### Ethical consideration

The study was ethically approved by the Institutional Review Board of Aklilu Lemma Institute of Pathobiology, Addis Ababa University and a supportive letter was obtained from Tigray Regional State Health Bureau and Alamata District Health Office. Moreover, the aim of the study was explained to the study participants and written and verbal consent was obtained. Those individuals who were found positive for *S.mansoni* infection were treated with praziquantel (40 mg/kg body weight) according to the WHO guideline without incurring any cost on the study individuals. Similarly those who were found positive for soil-transmitted helminths were treated with albendazole (single dose of 400 mg).

### Data analysis

A data-base was developed to store quantitative data using Microsoft Office Excel 2007 spreadsheets and imported to statistical software. STATA version 11 was used to compute descriptive statistics of variables collected during the study. An independent t-test was carried out to compare the mean intensity of schistosomiasis among male and female study individuals. One way ANOVA was also used to compare the differences in intensity among age groups of the study individuals. P-value <0.05 was reported as statistically significant.

## Results

### Demographic characteristics of the study individuals

A total of 371 individuals (male = 211 and female = 160) from three communities participated in this study. The majority of them (68.2%) were residents of semi-urban (Waja and Timuga) settings and the remaining individuals were from Aroresha, which is a rural type of setting. The mean age of study individuals was 22.2 (sd = 15.7) years.

#### *Schistosoma mansoni* results

**Figure 2:**
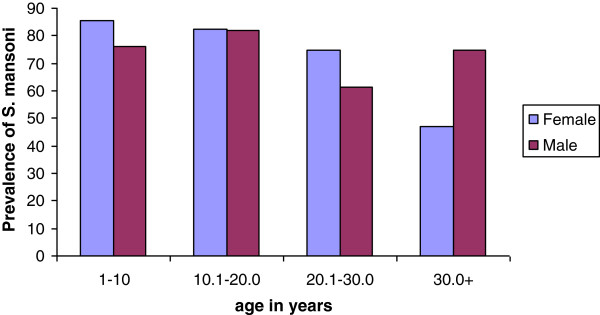
**Prevalence of *S. mansoni *** **by sex, Waja-Timuga, Dec. 2011-Mar. 2012.**

The mean intensity of *S. mansoni* infection among study participants in Waja-Timuga was 234 (sd = 431) EPG. There was a statistically significant difference (p < 0.001) in intensity of *S.mansoni* infection between females (mean EPG = 174, sd = 261) and males (mean EPG = 279, sd =521) (Figure 2). However, there was no statistically significant difference in the mean EPG of rural and urban residents (140.0 versus 169.6).

**Figure 3:**
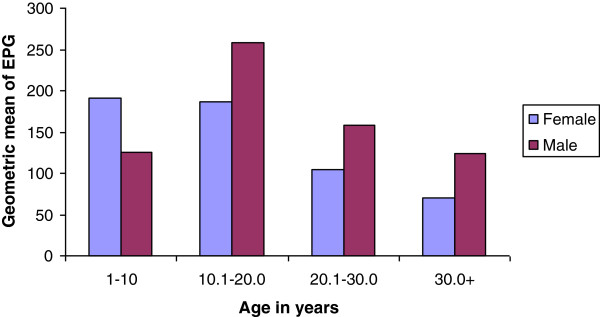
**Geometric mean as a measure of intensity of infection of *S. mansoni *** **by sex, Waja-Timuga, Dec. 2011-Mar. 2012.**

A significant difference was observed between the intensity of *S.mansoni* infection between residents of Waja and Aroresha areas (×^2^ = 33.7, p < 0.001). In the Waja area 27%, 19% and 19% of the study individuals had light, moderate and heavy infections, respectively. Among study individuals recruited from the Timuga area and of those infected, 29.4% had light infection, 19% had moderate infection and 19.6% had heavy infection. On the other hand, in the Aroresha area 32.2%, 42.4% and 14.4% had light, moderate and heavy infections, respectively. Intensity of infection did not significantly differ between sexes (P = 0.218) (Figure 3).

The highest intensity of *S.mansoni* infection was observed in the age range of 10.1-20.0 years followed by the age range of 1 –10 yeas (Table 1).

**Table 1:** **Summary of age specific intensity of *S.mansoni *** **infection, Waja-Timuga, Dec.2011-Mar.2012**

**Age category (years)**	**Level of Intensity of infection**			**Mean EPG ± SD**
	**Light**	**Moderate**	**Heavy**	
1 – 10	29.7%	35.6%	14.9%	222.4 ± 343.7
10.1 – 20	23.8%	25.4%	32.8%	361.3 ± 563.5
20.1 – 30	36.4%	21.8%	9.1%	165.8 ± 390.9
>30	33.3%	20.4%	6.5%	119.5 ± 273.3

### Ultrasonography

Of all the individuals (n = 371) participating in parasitological screening, only 162 individuals were examined using ultrasonography for detection of schistosomal periportal fibrosis.

PPF status by parasitological characteristics is shown in Table 2. Final PPF status of study individuals was determined using a combination of results of image pattern ultrasound and PBWT. Based on this, over all prevalence of PPF status was 12.3%. Presence/absence of ova of *S.mansoni* were not significantly associated with PPF status (p = 0.238). Among egg positive individuals, 54.9% had no PPF, 8.0% had indeterminate PPF and 11.1% had definite PFF.

**Table 2:** **Categories of PPF by parasitological characteristics of *S.mansoni *** **infection, Waja-Timuga, Dec. 2011- Mar. 2012**

***S*. *mansoni *** **infection**	**Category of PPF based on image pattern and PBWT-for-height**			
	**No PPF (%)**	**Indeterminate PPF (%)**	**Definite PPF (%)**	**Not graded (%)**
Positive	89 (54.9)	13 (8.0)	18 (11.1)	3 (1.9)
Negative	34 (21.0)	3 (1.9)	2 (1.2)	0 (0.0)
Total	123 (75.9)	16 (9.9)	20 (12.3)	3 (1.9)

**Figure 4:**
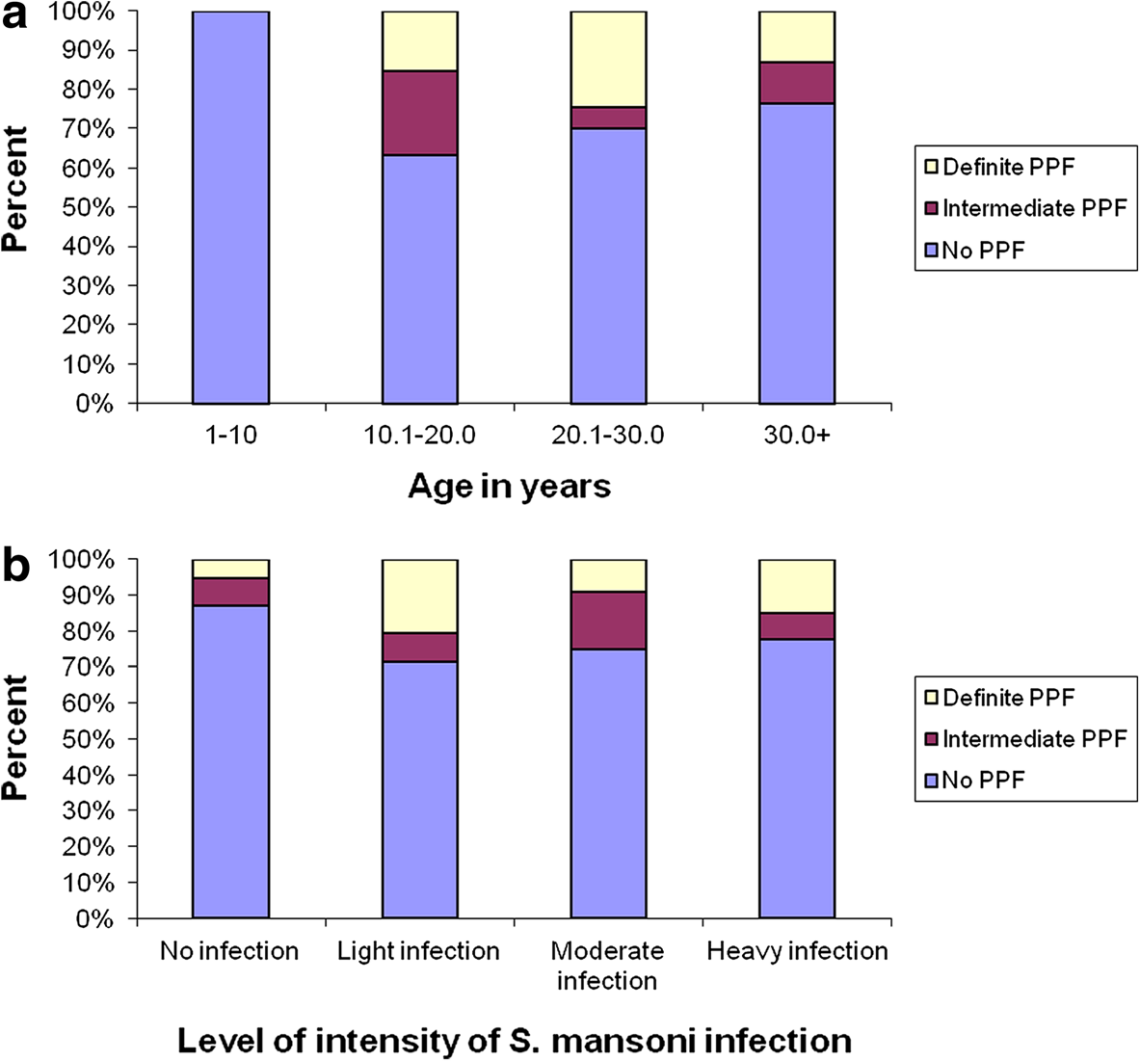
**Distribution of PPF by age (a) and intensity of *S. mansoni *** **infection (b), Waja-Timuga, Dec. 2011-Mar. 2012.**

Age and PPF status of the study individuals were significantly associated (p = 0.001). Among study individuals who had *S.mansoni* infection, prevalence of PPF had a sharp rise in the age range of 10.1 to 20 years and reached its peak in the age range of 20.1 to 30 years (Figure 4a). Whereas, statistical significant association was not observed (p = 0.297) between PPF status and intensity of infection (Figure 4b).

**Figure 5:**
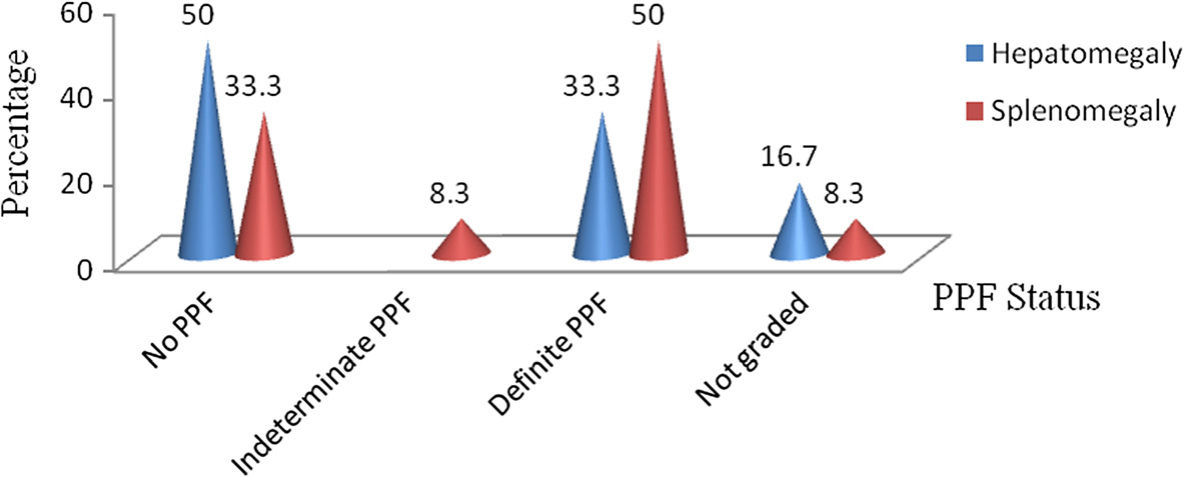
Prevalence of hepatomegaly and splenomegaly stratified by PPF status, Waja-Timuga, Dec. 2011-Mar. 2012.

Prevalence of hepatomegaly and splenomegaly (results obtained from ultrasonography) stratified by PPF status is summarized in Figure 5. The overall prevalence of hepatomegaly was 3.7% and that of splenomegaly was 7.4%. There was a demonstrable *S. mansoni* egg count in 83.3% and 91.7% of individuals who developed hepatomegaly and splenomegaly, respectively.

### Liver enzyme level test

Table 3 shows the proportion of study individuals with liver enzyme levels of SGOT and SGPT stratified by the categories of PPF status. Chemical analysis of SGOT and SGPT revealed that 8% (2/25) and 4% (1/25), respectively, were elevated from the normal ranges. Among the study individuals who had definite fibrosis 5.88% had elevated SGOT as well as SGPT. However, there was no statistically significant association between PPF status and liver enzyme level (p = 0.49).

**Table 3:** Summary of SGOT and SGPT by PPF status category, Waja-Timuga, Dec. 2011- Mar. 2012

**PPF status**^**i**^	**No. (%) SGOT level**^**ii**^		**No. (%) SGPT level**^**iii**^	
	**0**	**1**	**0**	**1**
**0**	3 (100)	-	3 (100)	-
**1**	4 (80)	1 (20)	5 (100)	-
**2**	16 (94.12)	1 (5.88)	16 (94.12)	1 (5.88)

^i^0 = no PPF, 1 = indeterminate PPF, 2 = definite PPF.

^ii^0 = within normal range, 1 = elevated.

^iii^0 = within range, 1 = elevated.

## Discussion

In the current study, prevalence of schistosomiasis mansoni was 73.9% and mean intensity of infection was 234 eggs per gram of stool. Based on the available evidence, the prevalence of *S. mansoni* infection varied greatly from one region to another [23-25]. Occupational difference is often reflected in schistosomiasis prevalence as occupational groups performing intensive contact with cercariae-infested water bodies have higher rates of infection [26,27]. Most previous community studies showed that males were more affected by schistosomiasis than females as the result differential frequency of contact with water bodies [28,29]. However, in the current study there was no significant difference in the prevalence of *S. mansoni* infection between males and females, which is also supported by some previous studies [16,30]. This could probably be due to similar occupations and water contact patterns to water bodies containing schistosome infected snails. This further also appeared to be attributed to analogous exposure in ecology, socioeconomic status of households, environmental sanitation, and water sources among other factors. All the three mean intensities in the present study sites were categorized under moderate schistosomiasis infection based on WHO [20] classification. The current finding reflects the general pattern of schistosomiasis in that only 17.8% of those infected are heavily infected but most people are infected moderately and/or lightly, the effects of which are thought to be minimal or ill defined [31].

In recently exposed communities, intensity of schistosomiasis infection increases with child age and then drops again in adulthood [32]. Reports from Jimma town, southwestern Ethiopia showed that intensity of *S. mansoni* infection peaked among individuals in the age ranges 10–19 years [30] and a similar finding was also reported in Kenya [33], both findings support the current study. These results can indicate that host maturity is an important aspect of resistance to schistosomiasis. Individuals living in areas of endemicity for *Schistosoma* species who have passed their midteen years generally have significantly less-intense infections than younger children, despite similar exposure to infectious parasites, suggesting that concomitant immunity develops with age in this case [34].

Morbidity due to schistosomiasis is caused by a granulomatous response to *S. mansoni* eggs deposited in peripheral portal veins that lead to hepatomegaly, portal hypertension and splenomegaly [35,36]. Previous studies have shown that the degree of morbidity associated with *S.mansoni* is related to intensity of infection [8,9] and Periportal fibrosis was also linked to infection intensity, as indicated by worm recovery on blood perfusion [37]. In the present study, significant association was observed between schistosomal PPF and intensity of infection. This finding is in line with earlier studies reported by [38,39]. An explanation for these results among individuals could be due to high exposures to potentially infected water bodies, which put the indicated age groups at higher risk of acquiring *S. mansoni* infection.

The proportion of individuals with hepatomegaly decreased and splenomegaly increased with advancing stages of PPF. On the other hand, Berhe *et al*. [39] reported that prevalence of hepatomegaly and splenomegaly varied significantly by different back ground characteristics. The variability of these results might be due to the similarity in gender-related behavioral and occupational exposures to potentially infected water bodies in the present study area.

Mild to moderate elevations of the liver enzymes are common. Fatty liver, chronic hepatitis B and C and long time use of alcohol are among the cause of mild to moderate liver enzyme elevation [40]. In the current study, most individuals who had periportal fibrosis showed liver enzymes within the normal range. This could probably be due to the small number of study individuals employed in the tests. Moreover, the precise levels of these enzymes do not correlate well with the extent of liver damage or the prognosis. Because these enzymes will be found in blood when there is parenchyma damage and/or in late stage of infection when there is decompensate of liver and presence of co-infection, on the other hand, the study individuals were apparently healthy. Thus, the exact levels of SGOT and SGPT levels cannot be used to determine the degree of liver disease or predict the future [40], however, they are crucial as bio markers of liver function test [41].

## Conclusion

The high prevalence of *Schistosoma mansoni* infection and schistosomal periportal fibrosis observed in the study area calls for a periodic deworming program to reduce transmission, worm burden and morbidity. Mass praziquantel administration should be complemented with improved sanitation and access to clean water, together with appropriate health education and environmental measures, which are required in order to produce a sustainable impact on transmission of the disease.

## Abbreviations

CSA: Central statistical agency of Ethiopia; EPG: Eggs per gram; IU: International unit; L: Liter; Masl: Meter above sea level; PBWT: Portal branch wall thickness; PPF: Periportal fibrosis; SD: Standard deviation; SGOT: Serum glutamic-oxaloacetic transaminase; SGPT: Serum glutamate-pyruvate transaminase; WHO: World Health Organization.

## Competing interests

The authors have no competing interests. Hence I, the undersigned, declare that this thesis is my original work and has not been presented for a degree in any other university and that all sources of materials used for the thesis have been duly acknowledged.

Investigator’s name: Nigus Abebe Shumuye.

## Authors’ contributions

Dr Nega Berhe carried out ultrasonographic examinations, participated in the design and coordination of the study and in proof reading of the manuscript. Prof. Berhanu Erko participated in design and coordination of the study and in proof reading of the manuscript. Dr Girmay Medhin participated in the study design and performed the statistical analysis and in proof reading of the manuscript. All authors read and approved the final version of the manuscript.

## Authors’ information

Nigus Abebe (BSc, MSc) College of Veterinary Medicine, Mekelle University.

● Lecturer at Mekelle University.

● Interested and working in the area of parasitology and infectious diseases such as parasitic diseases, vector borne diseases, zoonotic diseases.

Nega Berhe (MD, PhD) Aklilu Lemma Institute of Pathobiology, Addis Ababa University.

→Clinical researcher at Addis Ababa University.

→Working in the areas of Visceral Leishmaniasis, Schistosomiasis mansoni, HIV Infections, Endemic Diseases and AIDS-Related Opportunistic Infections.

Berhanu Erko, Professor of Medical Parasitology, Aklilu Lemma Institute of Pathobiology, Addis Ababa University.

→Working in areas of parasitology such as Schistosomiasis mansoni, Helminthiasis, Intestinal Parasitic Diseases, Ascariasis and Falciparum Malaria.

Girmay Medhin (BSC, MSc, PhD) Bio-Statistician, epidemiologist, Aklilu Lemma Institute of Pathobiology, Addis Ababa University.

→Interested and working in the area of Schistosomiasis mansoni, Mental Disorders, Schizophrenia, Helminthiasis and Latent Tuberculosis.

## Supplementary Material

Additional file 1 Standard images used during ultrasonography (Berhe et al., 2007). Click here for file
