# Supplementary material for: Clinico-epidemiological study of Schistosomiasis mansoni in Waja-Timuga, District of Alamata, northern Ethiopia
Source: Parasit Vectors. 2014 Apr 1;7:158. doi: 10.1186/1756-3305-7-158 (PMC4022361; doi:10.1186/1756-3305-7-158)
Supplement: Additional file 1 — Standard images used during ultrasonography (Berhe et al., 2007). [file 1756-3305-7-158-S1.doc]

Additional file 1: Standard images used during ultrasonography (Berhe et al., 2007)

Normal portal vessels


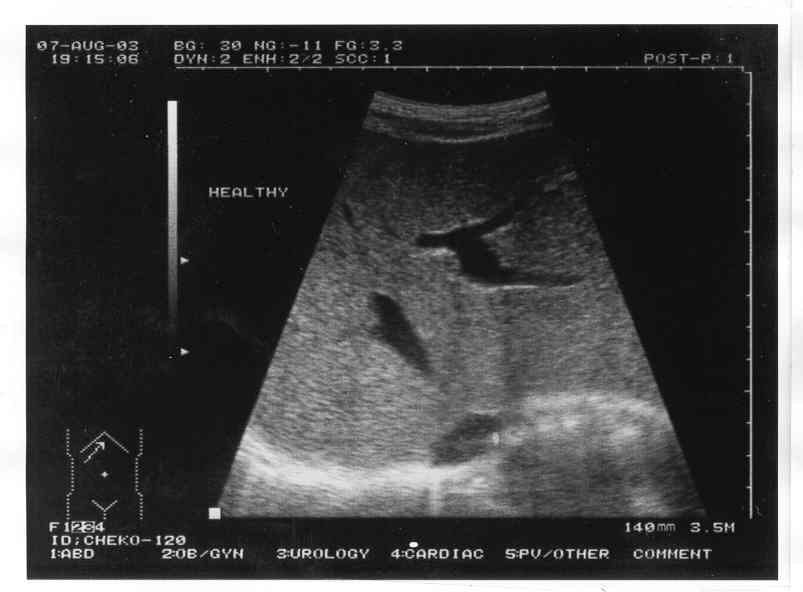


Periportal fibrosis


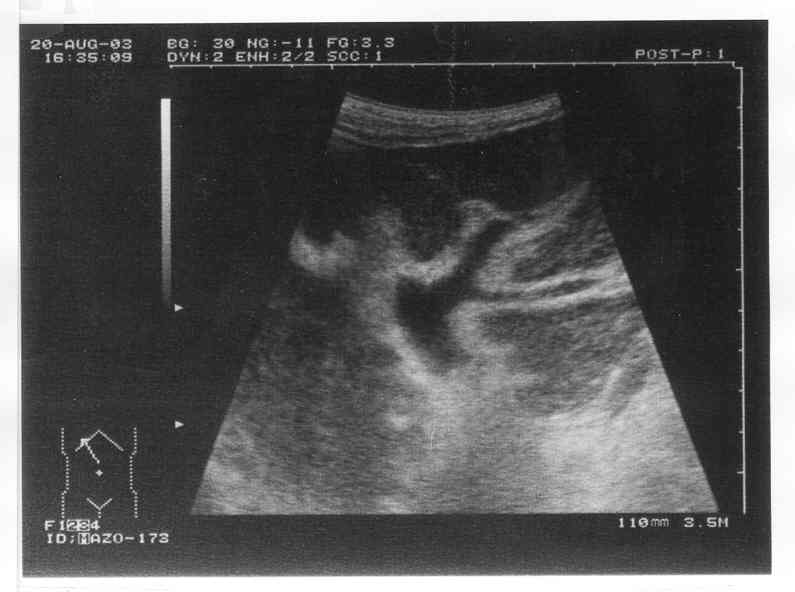


Schistosomal Periportal Fibrosis (Berhe *et al.,* 2007)
